# Respiratory Effects of Sarafotoxins from the Venom of Different *Atractaspis* Genus Snake Species

**DOI:** 10.3390/toxins8070215

**Published:** 2016-07-11

**Authors:** Stéphanie Malaquin, Sam Bayat, Osama Abou Arab, Gilles Mourier, Emmanuel Lorne, Saïd Kamel, Hervé Dupont, Frédéric Ducancel, Yazine Mahjoub

**Affiliations:** 1 Service de Réanimation Polyvalente, Pôle d’Anesthésie Réanimations, CHU Amiens F-80054 Amiens cedex, France; malaquin.stephanie@chu-amiens.fr (S.M.); Abouarab.osama@chu-amiens.fr (O.A.A.); lorne.emmanuel@chu-amiens.fr (E.L.); dupont.herve@chu-amiens.fr (H.D.); 2 Unité INSERM U1088, University of Picardie Jules-Verne, CURS site CHU, F-80054 Amiens cedex, France; Kamel.said@chu-amiens.fr; 3 Unité INSERM U1105, University of Picardie Jules-Verne, CURS site CHU, F-80054 Amiens cedex & Explorations Fonctionnelles Pédiatriques, CHU Amiens F-80054 Amiens cedex, France; Sam.Bayat@hcuge.ch; 4 CEA, iBiTec-S, Service d’Ingénierie Moléculaire des Protéines (SIMOPRO), CEA Saclay, F-91191 Gif sur Yvette, France; Gilles.MOURIER@cea.fr; 5 CEA, iMETI, Service d’Immunologie des Infections Virales et des Maladies Auto-Immunes (U1184), CEA Fontenay-aux-Roses, F-92265 Fontenay-aux-Roses, France; frederic.ducancel@cea.fr

**Keywords:** *Atractaspis*, long sarafotoxins, respiratory mechanics, forced oscillation technique, hysterisivity

## Abstract

Sarafotoxins (SRTX) are endothelin-like peptides extracted from the venom of snakes belonging to the Atractaspididae family. A recent in vivo study on anesthetized and ventilated animals showed that sarafotoxin-b (SRTX-b), extracted from the venom of *Atractaspis engaddensis*, decreases cardiac output by inducing left ventricular dysfunction while sarafotoxin-m (SRTX-m), extracted from the venom of *Atractaspis microlepidota microlepidota*, induces right ventricular dysfunction with increased airway pressure. The aim of the present experimental study was to compare the respiratory effects of SRTX-m and SRTX-b. Male Wistar rats were anesthetized, tracheotomized and mechanically ventilated. They received either a 1 LD_50_ IV bolus of SRTX-b (*n* = 5) or 1 LD_50_ of SRTX-m (*n* = 5). The low-frequency forced oscillation technique was used to measure respiratory impedance. Airway resistance (Raw), parenchymal damping (*G*) and elastance (*H*) were determined from impedance data, before and 5 min after SRTX injection. SRTX-m and SRTX-b injections induced acute hypoxia and metabolic acidosis with an increased anion gap. Both toxins markedly increased Raw, *G* and *H*, but with a much greater effect of SRTX-b on *H*, which may have been due to pulmonary edema in addition to bronchoconstriction. Therefore, despite their structural analogy, these two toxins exert different effects on respiratory function. These results emphasize the role of the *C*-terminal extension in the in vivo effect of these toxins.

## 1. Introduction

Sarafotoxins (SRTXs) extracted from the venom of snakes belonging to the *Atractaspis* genus and endothelins synthesized by mammalian endothelial cells belong to the same family of endothelin-like peptides [1,2]. Sarafotoxin-b (SRTX-b), extracted from the venom of *Atractaspis engaddensis,* is a 21-amino-acid-long peptide that interacts with endothelin receptors (ET-A and ET-B) situated on the membrane of various cells [3]. Recently, SRTX-m has been discovered in the venom of *Atractaspis microlepidota microlepidota* [4]. This peptide has a longer *C*-terminus extension with three additional residues “D-E-P”. SRTX-b and SRTX-m have a high sequence homology (more than 65%) and have the same three-dimensional conformation: an extended structure of the four *N*-terminal amino acids, a bend between positions +5 and +8, and an alpha-helical conformation of the Lys9-Cys-15 segment [5]. The *C*-terminus extension of SRTX-b (residues +16/+21) is conformationally flexible and disordered, while that of SRTX-m is more restricted [6]. It has been demonstrated that the *C*-terminus extension of these toxins determines the affinity and selectivity towards endothelin receptors, as SRTX-m shows a very low affinity for ET-A and ET-B human receptors compared to SRTX-b [6,7]. Nevertheless, the toxicity as assessed by the LD_50_ of these two sarafotoxins is in the same range (15 μg/g vs. 30 μg/g for SRTX-m). A previous in vivo animal study showed that SRTX-b and SRTX-m have very different cardiovascular effects: SRTX-b induces left ventricular dysfunction, resulting in decreased cardiac output, while SRTX-m induces right ventricular dysfunction and increased airway pressure [8]. However, the precise effects of SRTX-m on respiratory mechanics and gas exchange have not been investigated yet. The aim of the present study was to assess the effects of both sarafotoxins on respiratory function. We used the forced oscillation technique to compare the differential effects of these toxins on airway and lung tissue mechanical parameters.

## 2. Results

**Figure 1:**
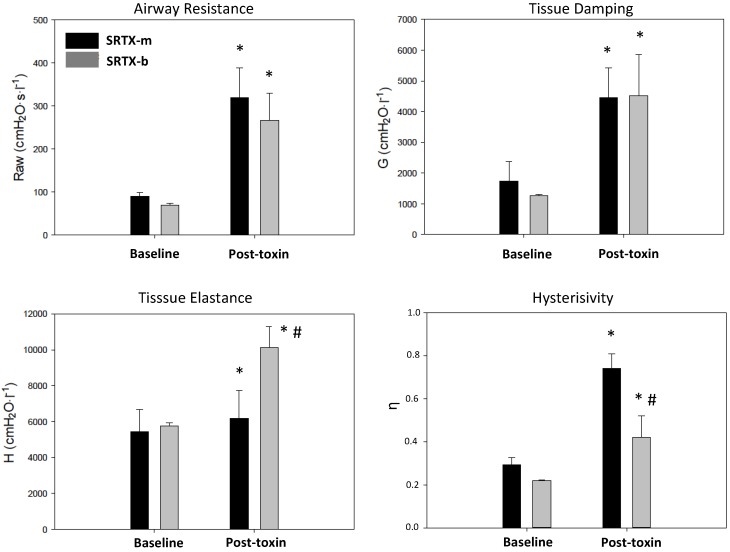
Respiratory mechanics parameters; data are presented as mean ± SD, *n* = 5; *: *p* < 0.05 vs. Baseline; #: *p* < 0.05 vs. SRTX-m, by two-way repeated measures ANOVA.

Airway resistance (Raw) and tissue damping (*G*) were significantly increased in both groups after 5 min (Figure 1). Tissue elastance (*H*) increased significantly in both groups; however, this effect was significantly greater in the SRTX-b group. Frothy fluid was also present in the tracheal cannula at the end of the experiment in this group. Lung hysteresivity (η) was significantly increased in both groups at 5 min. This parameter was significantly higher in the SRTX-m group.

In both groups, PaO_2_ was significantly decreased 5 min after toxin challenge (Table 1), despite alveolar hyperventilation. Bicarbonate levels were significantly decreased in both groups, while the anion gap was significantly increased after injection. Serum chloride was significantly increased in both groups (Table 1).

## 3. Discussion

In this study we investigated the impact of SXTR-b and SRTX-m IV bolus injection on respiratory mechanical properties using the low frequency forced oscillation technique. We found both similarities and differences in the effects of the two toxins on respiratory mechanics and gas exchange.

The marked increase in Raw following SRTX-m and SRTX-b injections was likely due to acute bronchoconstriction. Previous studies have suggested that the bronchoconstrictor effect of endothelins and ET-like toxins may be mediated via ET-B receptors on the airway smooth muscle [9,10]. Intravenous injection of SRTX-6c, an ET-B selective agonist, induced a significant increase in airway resistance in the pig [11]. This effect was inhibited by IV administration of bosentan, a non-selective endothelin receptor blocker. Other mechanisms may have participated in the acute bronchoconstriction observed after SRTX injection such as parasympathetic activation via a reflex vagal mechanism, epithelial release of cyclooxygenase products [12] or airway wall thickening due to peribronchial edema [10,13].

Endothelins play an important role in the pathogenesis of many obstructive airway diseases characterized by bronchoconstriction, mucous hyperplasia, airway remodeling and inflammation [14]. ETs produce their effect by acting via two established types of receptors, namely ET-A and ET-B. The pulmonary vascular endothelium is the richest source of ET in the body. The lung is the primary organ of ET metabolism and clearance [15]. Previous studies have shown that airway smooth muscle cells express ET-B and ET-A receptors and that agonists acting on these receptors produce a bronchoconstrictor effect [14]. A low affinity of SRTX-m for vascular smooth muscle cell ET-A receptors may explain its relatively lower vasoconstrictor effect on pulmonary vessels, and may facilitate the access of this toxin to airway ET receptors, predisposing the airway to bronchoconstriction [16,17]. This phenomenon may be enhanced by the higher affinity of SRTX-m for ET-B receptors compared to ET-A receptors [6].

In the SRTX-m group, the increase in airway resistance was associated with marked elevations in *G* and η and a moderate but significant increase in *H*. SRTX-b, on the other hand, had a much greater effect on *H*. Effects of ET-1 on respiratory mechanics have been previously studied by Nagase et al. in mice using the alveolar capsule technique [10]. In their study, ET-1 induced significant increases in airway and tissue resistance as well as *H* and η. These findings are consistent with our SRTX data. Moderate elevations in *H*, *G* and η, the ratio of dissipative and elastic processes in the lung parenchyma [18], may be the direct consequences of airway narrowing. Airway closure and volume losses lead to an increase in both *H* and tissue Raw [19,20]. On the other hand, bronchoconstriction can significantly contribute to parenchymal heterogeneities, leading to artefactual increases in *G* [21]. Finally, η itself may have been modified by the putative effect of SRTX on parenchymal viscoelasticity [22].

Although the above mechanisms may explain moderate elevations in *H*, *G* and η, the much greater increases in *H* observed with SRTX-b are likely to be due to changes in the parenchymal tissue properties. Isolated rat lung experiments suggest that endothelins can strongly induce lung weight gain via increased transcapillary fluid filtration [9]. Data from these experiments suggest that increased edema formation in this model is the result of potent pulmonary venoconstriction, mediated by ET-A receptors, which results in marked increases in pulmonary microvascular pressure [9]. Since SRTX-b shares structural and functional homology with ET-1, its intense effect on pulmonary elastance in the present study may have been the consequence of pulmonary edema. Moreover, in another study, in isolated, ventilated, perfused rat lung, ET-1 caused a net accumulation of alveolar fluid, increased pulmonary capillary pressure, decreased perfusate flow and accelerated lung weight gain [23]. These results suggest that ET-1, by elevating pulmonary microvascular pressure, contributes to pulmonary edema formation. Corroborating this hypothesis in our experiments, we observed abundant and frothy fluid in the tracheal cannula after SRTX-b injection, suggesting the presence of acute pulmonary edema. Another mechanism that may have further contributed to the development of hydrostatic pulmonary edema following SRTX-b injection in the present study was left ventricular failure. This hemodynamic phenomenon cannot be evaluated, as left ventricular pressure was not assessed in this protocol. However, in a previous experimental study focusing on the hemodynamic effects of SRTX-b, we demonstrated that SRTX-b exerted a negative lusitropic effect, i.e., impairment of left ventricular (LV) relaxation reflected by an increase of Tau, the relaxation time constant [8]. This finding was in accordance with previous animal and human studies [24]. We speculate that an impairment of LV relaxation may have induced elevations in pulmonary microvascular hydrostatic pressure, leading to edema, which would explain the marked increase in tissue elastance in this study. Furthermore, lung hydrostatic edema may have contributed to the elevation of Raw [13]. Moreover, the possibility that SRTX-b may have increased the permeability of the capillary-alveolar barrier, thereby contributing to pulmonary edema formation, cannot be excluded and further studies are needed to elucidate this point.

Blood gas analysis revealed marked disturbances in gas exchange and acid-base equilibrium following SRTX injection in both groups. Acute hypoxemia was probably initially induced by acute bronchoconstriction in both groups, compounded by suspected acute pulmonary edema in the SRTX-b challenge. Hypoxemia was associated with metabolic acidosis as suggested by a decrease in HCO3^−^. The increased anion gap may have been due to an increase in blood lactates induced by hypoxia. As the ventilatory settings were not modified during the experiment, decreases in PCO_2_ after SRTX challenge may be explained by an early and marked decrease in cardiac output [11], decreasing carbon dioxide transport to the lung.

SRTX-b and SRTX-m exerted different effects on respiratory mechanics in this study. These different effects could be due to the presence of a *C*-terminus extension with a different spatial conformation between the two toxins. Further experimental studies including different SRTX challenges with specific ET receptor subtype inhibitors are warranted in order to assess this hypothesis.

This study presents several limitations. First, due to the rapid effects of both toxins, cardiac function could not be evaluated concomitantly to respiratory mechanical changes. However, since these effects have been demonstrated in a previous study [11], we feel confident that they were reproduced here. Second, blood lactate was not measured due to a technical limitation of the blood gas analysis apparatus. This would have been helpful in establishing the cause of the elevated anion gap, and should be included in further studies. Third, we did not study the dose-response relationship for SRTX. We used only one dose of 1 LD_50_ as in previous studies in order to have a comparable effect [8]. Finally, SRTX-m was initially reported to have been isolated from the venom of *Atractaspis microlepidota microlepidota*, and that finding was confirmed by molecular cloning from the corresponding venom-gland cells [4]. However, as a result of subsequent and posterior taxonomic findings [25], the precise identity of the parental snake appears less clear and the toxin may have been isolated from another species, such as *Atractaspis watsoni*, *Atractaspis micropholis* or *Atractaspis fallax*. Consequently, subsequent researchers seeking to reproduce the results described here with venom obtained from snakes rather than in vitro synthesis should bear this taxonomic uncertainty in mind. Nevertheless, the primary sequence of SRTX-m is unambiguous and will be the basis of any reproduction and/or further exploration of the biological properties associated with that new family of long-sarafotoxins.

## 4. Conclusions

To the best of our knowledge, this is the first study that assessed the in vivo respiratory effect of SRTXs. The short SRTX, SRTX-b, and the long SRTX, SRTX-m, seem to act in different ways on respiratory mechanics. Both agents had potent bronchoconstrictive effects. SRTX-b induced larger increases in lung tissue elastance that may have been due to pulmonary edema. These results emphasize the major role of the *C*-terminal extension of these peptides in determining the in vivo respiratory effects of endothelin-like peptides. By providing new insights into the mechanisms by which endothelin-like peptides exert their in vivo effect, this study may contribute to better understanding the cardiac and pulmonary diseases in which “the endothelin system” is involved (i.e., pulmonary hypertension, asthma or heart failure).

## 5. Materials and Methods

### 5.1. Peptide Synthesis

SRTX-b was purchased from Sigma (St Quentin Fallavier, France). SRTX-m was synthesized by automated chain assembly with a standard Applied Biosystems 433 peptide synthesizer, as previously described [6,7]. Sarafotoxin-m was synthesized on a Fmoc-Trp(Boc)-wang resin by standard solid phase synthesis techniques. Cysteines were introduced as Fmoc-Cys(Trt)-OH. Dicyclohexylcarbodiimide and 6-Chloro-1-hydroxybenzotriazole were used as coupling reagents. Peptides were separated from the resin after A TFA deprotection. For each peptide, the crude material was purified by HPLC (Waters instruments, Guyancourt, France) using a C18 column with an 18%–30% CH_3_CN gradient in 0.1% TFA in water. Peptide reduced forms were subjected to an oxidative reaction in 0.1 M Tris/1 mM EDTA buffer containing 0.5–2 M guanidine hydrochloride in the presence of reduced (GSH) and oxidized (GSSG) glutathione in a molar ratio of 1/10/100 in peptide/GSSG/GSH at a peptide concentration of 0.05 mg/mL [6,7]. After 36 h at 4 °C, oxidized forms of the toxins were purified by HPLC and characterized by amino acid analysis using an automatic analyzer (Applied Biosystem 130 A) and mass spectrometry on a Nermag spectrometer coupled to an analytical electrospray source.

### 5.2. Animal Preparation

Six-week-old Male Wistar rats weighing between 210 g and 340 g were maintained in a temperature- and humidity-controlled room with 12:12 h light-dark cycle. They were given standard chow and were fasted for 12 h before the experiment with ad libitum access to water. Animal experiments were performed in accordance with the recommendations of the EU and the French National Committee for the care and use of laboratory animals. The institutional Animal Care Ethics committee of Amiens school of Medicine (CREMEAP No. 161112 approved on 16/12/2012) approved the experimental protocol. Anesthesia was induced by placing the animals in a chamber containing 3% isoflurane (Furane, AbbVie, Rungis, France). Animals were then tracheotomized and mechanically ventilated with an FiO_2_ of 21% to limit the development of pulmonary hyperoxic atelectasis. Tidal volume was 6.2 × M^1.01^ mL and the respiratory rate was 53.5 × M^−0.26^·min^−1^ (M = animal weight in kg) [26]. Anesthesia was maintained by inhaled isoflurane (1%–1.5%) and analgesia was ensured by intraperitoneal injection of 1 mg/kg of morphine (Morphine, Aguettant, Lyon-Gerland, France). Adequate anesthesia/analgesia was regularly checked by the lack of response to tail pinch and 20% of the initial dose of morphine was injected every 40 min. When the animal was adequately anaesthetized, 2 mg/kg of pancuronium bromide was injected IP to induce muscle relaxation. Body temperature was maintained at 37–37.5 °C with a heating pad. A fluid filled catheter (Neoflon 26 G, Becton Dickinson, Helsingborg, Sweden) was inserted in the right carotid artery for blood pressure monitoring and blood gas analysis (Radiometer, ABL77, Copenhagen, Denmark). A second catheter was introduced in the left jugular vein for injections.

### 5.3. Measurement of Respiratory Mechanics

We used the low-frequency forced oscillation technique (FOT), in order to precisely separate airway and lung tissue contributions to the total respiratory system impedance, using the constant-phase model [27,28]. Two sighs (inflation to 30 cm H_2_O) were delivered before the beginning of data collection. At end-expiration, mechanical ventilation was paused and a small-amplitude (1 cm H_2_O peak-to-peak) forcing signal was delivered into the respiratory system by a loudspeaker-in-box system connected to the tracheal cannula via a polyethylene tube (100 cm length, 2.0 mm inner diameter). All measurements were performed at zero end-expiratory pressure. The loudspeaker was driven by a computer-generated pseudorandom signal ranging from 0.5 to 21 Hz. Lateral pressures were measured at the loudspeaker end (*P*_1_) and the distal end (*P*_2_) of the wave-tube with miniature sidearm transducers (ICS 33NA00D). These pressure signals were low-pass filtered (<25 Hz) and digitized at a sampling frequency of 128 Hz. The pressure transfer function (*P*_1_/*P*_2_) was created by fast Fourier transformation from the 8 s recording. The input impedance of the respiratory system (*Z*rs) was computed from the pressure transfer function as the load impedance of the wave-tube [19] by using the transmission line theory [29]:

*Z*rs = Z_0_·sinh(γ*L*)/[*P*_1_/*P*_2_ − cosh(γ*L*)] where *L* is the length of the wave tube, Z_0_ is the characteristic impedance of the wave tube, γ is the complex propagation wave number, sinh is hyperbolic sine and cosh is hyperbolic cosine. Both Z_0_ and γ depend on the geometrical parameters of the wave tube (diameter, material constants and gas inside the tube). Three to five *Z*rs spectra were ensemble-averaged at baseline and 5 min after toxin challenge. A model that includes Newtonian resistance (Raw), inertance (Iaw) in series with constant-phase tissue compartments incorporating tissue damping (*G*) and elastance (*H*) was fitted to the averaged *Z*rs data [27]. (Hysterisivity (η) was calculated as the *G*/*H* ratio. The data at frequencies coinciding with heart rate and its harmonics were often corrupted (as evidenced by poor coherence and a high SD) and they were omitted from the model fitting.

### 5.4 Experimental Protocol

Five rats were allocated to the SRTX-b group and five rats were allocated to the SRTX-m group. A 15 min stabilization period was observed after completion of the surgical preparation. Animals were randomly allocated to two groups to receive SRTX-b (SRTX-b group), or SRTX-m (SRTX-m group). Each toxin was administered at a dose of 1 LD_50_: 15 µg·kg^−1^ for SRTX-b, 32 µg·kg^−1^ for SRTX-m [4,30]. All doses were diluted in 1 mL of saline and injected via the left jugular vein over 1 min. All respiratory measurements were performed at baseline and 5 min after injection of the toxin.

### 5.5 Statistical Analysis

The scatter of the data was expressed as the standard deviation (SD). The Kolmogorov-Smirnov test was used to test data for normality. Student’s *t*-test was used to test differences between baseline and post-challenge gas exchange data. Two-way repeated measures analysis of variances (ANOVA), with variables of toxin type (SRTX-m and SRTX-b) and experimental condition (Baseline, Challenge) was used to evaluate the changes in the respiratory mechanical parameters. Pairwise comparisons were performed by using Student-Newman-Keuls multiple comparison procedures. Each test was performed with a significance level of *p* < 0.05.

## Figures and Tables

**Table 1:** Blood gas analysis. Data are presented as mean ± SD, *n* = 5; *: *p* < 0.05 vs. Baseline. AG = anion gap.

Toxin	pH	PaO_2_ (mmHg)	PaCO_2_ (mmHg)	HCO_3_^−^ (mmol·L^−1^)	Cl^−^ (mmol·L^−1^)	AG (mmol·L^−1^)
**Baseline**	7.43 ± 0.12	85 ± 21	25 ± 4	16 ± 5	113 ± 2	12 ± 4
**SRTX-m**	7.41 ± 0.14	65 ± 16 *	18 ± 5	12 ± 2	119 ± 3 *	17 ± 4 *
**Baseline**	7.51 ± 0.04	100 ± 18	20 ± 5	16 ± 3	116 ± 2	12 ± 2
**SRTX-b**	7.49 ± 0.18	70 ± 25 *	13 ± 7 *	9 ± 2 *	12 ± 4 *	19 ± 3 *

## Abbreviations

The following abbreviations are used in this manuscript

SRTXs	sarafotoxins
SRTX-b	sarafotoxin b extracted from the venom of *Atractaspis engaddensis*
SRTX-m	sarafotoxin m extracted from the venom of *Atractaspis microlepidota microlepidota*
ET	endothelin
ET-A	endothelin-receptor subtype A
ET-B	endothelin-receptor subtype B
Iaw	inertance
Raw	resistance
*G*	damping
*H*	elastance
η	hysterisivity
